# bc-GenExMiner 4.5: new mining module computes breast cancer differential gene expression analyses

**DOI:** 10.1093/database/baab007

**Published:** 2021-02-18

**Authors:** Pascal Jézéquel, Wilfried Gouraud, Fadoua Ben Azzouz, Catherine Guérin-Charbonnel, Philippe P Juin, Hamza Lasla, Mario Campone

**Affiliations:** Unité de Bioinfomique, Institut de Cancérologie de l’Ouest, Bd Jacques Monod, Saint Herblain Cedex 44805, France; CRCINA Team 8, UMR 1232 INSERM, Université de Nantes, Université d’Angers, Institut de Recherche en Santé-Université de Nantes, 8 Quai Moncousu—BP 70721, Nantes 44007, France; SIRIC ILIAD, Institut de Recherche en Santé-Université de Nantes, 8 Quai Moncousu-BP 70721, Nantes 44007, France; Unité de Bioinfomique, Institut de Cancérologie de l’Ouest, Bd Jacques Monod, Saint Herblain Cedex 44805, France; SIRIC ILIAD, Institut de Recherche en Santé-Université de Nantes, 8 Quai Moncousu-BP 70721, Nantes 44007, France; Unité de Bioinfomique, Institut de Cancérologie de l’Ouest, Bd Jacques Monod, Saint Herblain Cedex 44805, France; SIRIC ILIAD, Institut de Recherche en Santé-Université de Nantes, 8 Quai Moncousu-BP 70721, Nantes 44007, France; Unité de Bioinfomique, Institut de Cancérologie de l’Ouest, Bd Jacques Monod, Saint Herblain Cedex 44805, France; SIRIC ILIAD, Institut de Recherche en Santé-Université de Nantes, 8 Quai Moncousu-BP 70721, Nantes 44007, France; CRCINA Team 8, UMR 1232 INSERM, Université de Nantes, Université d’Angers, Institut de Recherche en Santé-Université de Nantes, 8 Quai Moncousu—BP 70721, Nantes 44007, France; SIRIC ILIAD, Institut de Recherche en Santé-Université de Nantes, 8 Quai Moncousu-BP 70721, Nantes 44007, France; Unité de Bioinfomique, Institut de Cancérologie de l’Ouest, Bd Jacques Monod, Saint Herblain Cedex 44805, France; SIRIC ILIAD, Institut de Recherche en Santé-Université de Nantes, 8 Quai Moncousu-BP 70721, Nantes 44007, France; CRCINA Team 8, UMR 1232 INSERM, Université de Nantes, Université d’Angers, Institut de Recherche en Santé-Université de Nantes, 8 Quai Moncousu—BP 70721, Nantes 44007, France; SIRIC ILIAD, Institut de Recherche en Santé-Université de Nantes, 8 Quai Moncousu-BP 70721, Nantes 44007, France; Oncologie Médicale, Institut de Cancérologie de l’Ouest—René Gauducheau, Bd Jacques Monod, Saint Herblain 44805, France

## Abstract

‘Breast cancer gene-expression miner’ (bc-GenExMiner) is a breast cancer–associated web portal (http://bcgenex.ico.unicancer.fr). Here, we describe the development of a new statistical mining module, which permits several differential gene expression analyses, i.e. ‘Expression’ module. Sixty-two breast cancer cohorts and one healthy breast cohort with their corresponding clinicopathological information are included in bc-GenExMiner v4.5 version. Analyses are based on microarray or RNAseq transcriptomic data. Thirty-nine differential gene expression analyses, grouped into 13 categories, according to clinicopathological and molecular characteristics (‘Targeted’ and ‘Exhaustive’) and gene expression (‘Customized’), have been developed. Output results are visualized in four forms of plots. This new statistical mining module offers, among other things, the possibility to compare gene expression in healthy (cancer-free), tumour-adjacent and tumour tissues at once and in three triple-negative breast cancer subtypes (i.e. C1: molecular apocrine tumours; C2: basal-like tumours infiltrated by immune suppressive cells and C3: basal-like tumours triggering an ineffective immune response). Several validation tests showed that bioinformatics process did not alter the pathobiological information contained in the source data. In this work, we developed and demonstrated that bc-GenExMiner ‘Expression’ module can be used for exploratory and validation purposes.

**Database URL**: http://bcgenex.ico.unicancer.fr

## Introduction

High-throughput gene expression data associated with their clinicopathological features represent a treasure trove of information for medical research. Hence, complex diseases such as cancer could benefit from this wealth of information. However, before it can benefit the widest possible range of researchers, these data and statistical mining functions need to be automated by bioinformatics experts, for instance in the form of integrated easy-to-use web-based tools.

‘Breast cancer gene-expression miner’ (bc-GenExMiner) is a disease-associated web portal launched in 2010 (http://bcgenex.ico.unicancer.fr) (1, 2). It offers the possibility to explore gene expression of genes of interest in breast cancer based on transcriptomic and clinicopathological data. Since the last publication presenting bc-GenExMiner v3.0, many additions and improvements have been made in the present version. In 2013, bc-GenExMiner v3.0 database included 3237 patient genomic data from 21 microarray studies, and two analysis modules were described: ‘Prognostic’ and ‘Correlation’. Currently, bc-GenExMiner v4.5 includes a total of 15 428 unique cases: 10 716 patient genomic data from 59 microarray studies and 4712 patient genomic data from three RNAseq studies. Furthermore, two non-tumour RNAseq genomic data are also included in this new version: healthy breast tissue, i.e. no history of cancer (*n* = 92), and tumour-adjacent tissue (*n* = 104). Here, we present the development of an ‘Expression’ analysis module, which permits 39 differential gene expression analyses, grouped into 13 categories, according to clinicopathological and molecular characteristics (‘Targeted’ and ‘Exhaustive’) and gene expressions (‘Customized’). Expression analyses can be performed by choosing microarray or RNAseq data. We hypothesize that concordance of the results based on these two kinds of data proves the robustness of gene expression investigations. Output results are visualized in the form of four types of plots: box and whisker, beeswarm, violin and raincloud.

One of the originalities of this new module, among others, is the possibility to explore gene expression according to the nature of the breast tissue. Three kinds of tissues are available: healthy (cancer-free), tumour-adjacent and tumour tissues. Therefore, it is possible to explore gene expression variations across tissues that are cancer free (healthy), more or less influenced by cancer cells (tumour-adjacent) and tumour. The latter can be split in function of eight criteria. Another originality is the possibility to explore gene expression in triple-negative breast cancer (TNBC) determined by immunohistochemistry (IHC) and/or basal-like intrinsic molecular subtype determined by PAM50, as well as in the three TNBC subtypes: C1, molecular apocrine tumours; C2, basal-like tumours infiltrated by immune suppressive cells and C3, basal-like tumours triggering an ineffective immune response (3–5).

One of the main issues in omics studies is the ‘short, fat data’ problem, i.e. too many more variables than observations (*p* >> *n*). In this case, statistical standard methods are difficult to apply. Notably, the likelihood of obtaining ‘false positives’ increases, not only in the identification of differentially expressed genes but also when building predictive models. One way to get around this problem is to increase the number of observations (patients). In practice, this consists in pooling many cohorts. But this approach requires a data normalization process. Unfortunately, this step can smooth and mitigate biological differences that are present in the different cohorts. That is why it is important to carry out validation tests after normalization in order to check whether the pathobiological information is still present in the pooled cohorts. To this end, numerous tests were conducted to ensure that ‘Expression’ module can be used for exploratory and validation purposes.

## Methods

### bc-GenExMiner v4.5 mRNA expression database

The following inclusion criteria were used to select data: microarray or RNAseq transcriptomic data, female breast cancer, invasive carcinomas, tumour macrodissection [no microdissection, no biopsy, except for The Cancer Genome Atlas (TCGA) cohort], no neoadjuvant therapy before tumour collection, minimum of 35 patients per cohort and no duplicate. On the latter point, data were filtered by sample identifier and by Pearson’s correlation analyses (*r* < 0.99). Available clinicopathological data linked to transcriptomic data were also retrieved. Healthy and tumour-adjacent data were added to this database version.

### Analyses

‘Expression’ module analysis includes three categories of analyses: ‘Targeted’, ‘Exhaustive’ and ‘Customized’.

#### Targeted and exhaustive expression analyses

Our aim was to develop programmed statistical analyses able to explore gene mRNA expression according to various clinicopathological and molecular characteristics: (i) nature of the tissue (healthy, tumour-adjacent and tumour); (ii) receptor statuses; (iii) nodal status; (iv) histological type; (v) Scarff–Bloom–Richardson (SBR) grade; (vi) Nottingham prognostic index (NPI); (vii) age; (viii) p53 status; (ix) intrinsic molecular subtypes; (x) basal-like (PAM50) and/or TNBC (IHC); (xi) TNBC subtypes and (xii) integrative clusters. Here, we will particularly focus on four categories of expression analyses according to nature of the tissue, p53 status, basal-like (PAM50) and/or TNBC (IHC), and TNBC subtypes.

##### Nature of the tissue.

Our objective was to propose gene expression exploration in three kinds of breast tissues at once: healthy (no history of cancer, i.e. reduction mammoplasty), tumour-adjacent and tumour. Furthermore, eight criteria are proposed to split tumours: oestrogen receptor (ER) (IHC), progesterone receptor (PR) (IHC), ER/PR combinations (IHC), HER2 (IHC), p53 (sequence-based), intrinsic molecular subtypes (PAM50), TNBC (IHC) and basal-like (PAM50). A set of gene expression signatures (GESs) was used for biological validation purpose. These GESs were categorized in the following four groups: (i) molecular subtyping: PAM50; (ii) metabolism evaluation: glycolysis and iron regulation [iron regulatory gene signature (IRGS)]; (iii) critical biological pathways in cancer: chromosomal instability (CIN), E2F3 transcription factor 3 (E2F3), MYC proto-oncogene BHLH transcription factor (MYC), perineural invasion (PNI), proliferation, transforming growth factor beta (TGFβ) and wound response; and (iv) prognostic: 38-GES, 70-GES and genomic grade index (GGI) (5).

##### p53 status.

As no gold standard exists, we offered the possibility to explore gene expression according to IHC, GES and sequencing modes of p53 status determination (6, 7). The *TP53* gene is the most frequently mutated gene in cancer, and downstream loss of p53 function promotes cancer. Mutated tumours are highly proliferative and trigger an immune response in breast cancer (8). In order to verify that this biological information was preserved irrespective of the method of p53 status determination and the nature of the data (i.e. microarrays or RNAseq), several biological tests were done relative to p53 GES, proliferation and immune response (representative, HLA and immune checkpoints) genes (5, 7–9).

##### Basal-like (PAM50) and/or TNBC (IHC).

These subtypes of tumours belong to the most aggressive breast cancers. TNBC status and basal-like molecular intrinsic subtype were determined by IHC and PAM50 GES, respectively (10). These two annotations are not equivalent (5, 11). For this reason, we proposed to explore gene expression in TNBC, in basal-like and in tumours that are both TNBC and basal-like. mRNA expression of forkhead box transcription factor C1 (*FOXC1*), probably the most discriminating gene between basal-like and other breast cancer intrinsic molecular subtypes, was used for biological validation purpose (12).

##### TNBC subtypes.

Several studies have shown that TNBC grouped into different subtypes characterized by marked biological features (3–5, 13, 14). Here, we chose to split TNBC into Jézéquel *et al.*’s TNBC subtypes because their integrative work was based on transcriptomic, IHC and proteomic data and was validated on external data. In short, C1 is composed of molecular apocrine tumours (or luminal androgen receptor); C2, the most aggressive subtype, is composed of basal-like tumours infiltrated by immune-suppressive cells and displays high neurogenesis activity; and C3 is also composed of basal-like tumours triggering an ineffective immune response, which is associated with high tumour-infiltrating lymphocytes and plasma cells, tertiary lymphoid structures and upregulation of immune checkpoints. TNBC subtyping method is described elsewhere (3). In order to validate this kind of analysis, expressions of eight TNBC subtype specific genes were explored: *AR* and *FOXA1* (C1: molecular apocrine genes); *FOXC1* (C2 and C3: basal-like gene); *CD8A, IGKC, STAT1, CD274* (*PD-L1*) and *PDCD1* (*PD1*) (C3: immune response and/or immune checkpoint genes).

#### Customized expression analysis

Another approach for differential gene expression analysis consists in splitting data by means of a splitting gene using different criteria (median, tertile, quartile, optimal and customized percentile) and comparing the expression of a gene of interest between these groups. The optimal criterion scans every splitting value with a range that goes from 20 percentile to 80 percentile and with a step of five percentiles. Four pairs of genes (tested gene and splitting gene) were tested. The first two ones were composed of correlated genes: *MKI67* and *AURKA*, which are considered as prototypic proliferation genes, and *GZMA* and *PRF1*, which are used to explore T-cell cytotoxicity (15). The last two ones were *GZMA* and *ESR1*, and *PRF1* and *ESR1*. ER-negative breast tumours (i.e. *ESR1*-low) are known to trigger immune response. ‘Quartile’ was used as splitting criterion, and microarray and RNAseq data sets were explored.

### Preprocessing

Non-Affymetrix® platform data were ratio-normalized or quantile-normalized, and Affymetrix® raw CEL files were MAS5-normalized for microarrays section, except for Affymetrix® Gene 1.0 ST CEL files (GSE36295 and GSE37751) which were RMA-gene-level normalized because MAS5 is inapplicable. With respect to RNAseq part, we used two publicly available data sets: TCGA breast cancer and Sweden Cancerome Analysis Network for breast cancer (SCAN-B). HTSeq-FPKM data for TCGA breast cancer samples were downloaded as gene expression profiles. They were processed following the mRNA analysis pipeline of TCGA (https://docs.gdc.cancer.gov/Data/Bioinformatics_Pipelines/Expression_mRNA_Pipeline). FPKM values were log2-transformed with an offset value of 0.1. In addition, we collected the log2(FPKM + 0.1) data set provided by authors and available from the NCBI Gene Expression Omnibus (GSE81538 and GSE96058) (16). Only for the part ‘Nature of the tissue’, RNAseq from both TCGA and GTEx were processed and normalized using the Rsubread package (17). TPM values were downloaded from GEO [GSM1536837 (tumour) and GSM1697009 (tumour-adjacent)] and FPKM from GSE86354 (Healthy), which were then converted to TPM in order to have homogeneous data. For only this part, all data sets were log2-transformed using an offset of 1. With the aim of merging studies data and creating pooled cohorts, methods for batch effect removal were performed. All studies data, except TNBC cohorts, were converted to a common scale (median at 0 and standard deviation at 1). For TNBC cohorts, ComBat method was used (18, 19).

### Statistical analysis

Welch’s and Dunnett–Tukey–Kramer’s tests were used for differential gene expression analyses. Chi-squared test was used to study the distribution of p53 mutation according to the nature of the data and p53 status determination method. *P*-values less than 0.05 were considered as statistically significant.

### Web tool architecture

bc-GenExMiner is based on a three-tier architecture. First one: human–machine interface requires a HTML browser with JavaScript enabled but does not need any particular visual plug-in tool. Second one: the Apache framework and PHP programming language with the support of the R software delivers the application layer. The R software manages all statistical calculations. A few external packages are used to perform specific tasks like plotting figures or using algorithms (data.table v1.12.2, devEMF v3.6-3, DTK v3.5). Third one: the MySQL relational database management system permits to store the patient cohorts as the data tier. Currently, 92 SQL tables are stored into the database—these were publicly downloaded from various sites (Gene Expression Omnibus, ArrayExpress and GDC portal for the most part).

### User interface

In an effort to develop an ‘easy-to-use’ web-based tool, a particular attention was paid to the user-friendliness of the graphic user interface. Analysis request is processed by PHP language and generates database extraction leading to statistical analysis with R and results presentation.

Reactive interface (using JavaScript and jQuery) was added to upgrade user experience. Moreover, to ensure the best graphical display, the scalable vector graphic image format (.svg) was implemented. Finally, users can download their favourite figure in two open-source formats: portable network graphics (png) and enhanced metafile (emf). This last file format permits lossless modifications as user’s wish within a freeware. Furthermore, to simplify results presentation, drop-down menus are proposed.

## Results

### bc-GenExMiner v4.5 mRNA database

This version includes annotated microarrays (59 cohorts; *n* = 10 716) and RNAseq (three cohorts; *n* = 4712) tumour transcriptomic data (Supplementary Table S1A and S1B; Supplementary Table S2A and S2B). Apart from tumour data, two other kinds of tissues were included: 92 healthy profiles, curated from GTEx database, and 104 tumour-adjacent patients from TCGA cohort.

### Targeted and exhaustive expression analyses

Thirty-eight differential gene expression analyses can be performed through ‘targeted expression analyses’ (one by one) or ‘exhaustive expression analyses’ (all at the same time): nature of the tissue (healthy breast tissue versus tumour-adjacent tissue versus tumour tissue) (*n* = 9); receptor statuses (*n* = 4); nodal status (*n* = 1); histological types (*n* = 1); SBR grade (*n* = 1); NPI (*n* = 1); age (*n* = 2); p53 status (*n* = 3); intrinsic molecular subtypes (*n* = 9); basal-like (PAM50) and/or TNBC (IHC) (*n* = 3); TNBC subtypes (*n* = 2); and integrative clusters (*n* = 2). Box and whisker, beeswarm, violin and raincloud plots are provided to visualize output results.

#### Nature of the tissue

**Figure 1.**
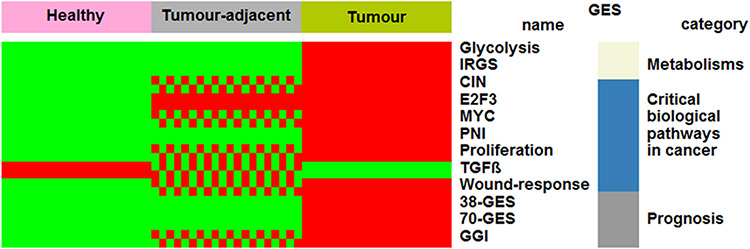
Comparisons of biological characteristics of the three breast tissues by means of GESs. The first row presents the three types of breast tissues. The other rows, from top to bottom, present significant GES scores in function of tissue type (green: low score; red and green checkerboard pattern: intermediate score; red: high score). This figure is an illustration of Supplementary Table S3.

Healthy and tumour-adjacent tissues were almost exclusively subtyped as luminal A (97%) by means of PAM50 GES (Table 1). This result is concordant with a previous study that reported that normal breast tissues clustered with luminal A subtypes (20). Biological characteristics of the three tissues were explored and compared by means of several GESs. Four profiles were identified: ‘healthy (H) < tumour-adjacent (TA) < tumour (T), for CIN, GGI, MYC, proliferation and wound response; ‘H < TA = T’ for E2F3; ‘H = TA < T’ for 38-GES, 70-GES, glycolysis, IRGS and PNI; ‘H > TA > T’ for TGFβ (Figure 1 and Supplementary Table S3). In the first case, increasing kinetics, which reflects the increase in biological aggressiveness, was observed. Healthy tissues displayed GES scores inferior to tumour-adjacent tissues, which themselves displayed GES scores inferior to tumour tissues. These results, together with E2F3, show that part of the biology of the tumour-adjacent tissue is influenced by the tumour or may contain isolated cancer cells. For this reason, it is important to distinguish healthy from tumour-adjacent tissues for differential gene expression analyses. On the contrary, five GESs did not show any difference between healthy and tumour-adjacent tissues. Only one decreasing kinetics profile was observed for TGFβ: H > TA > T. This profile might be explained by the fact that this multifunctional cytokine is known to show tumour-suppressive effects in normal mammary epithelium and in the early stage of breast cancer (21–23).

**Table 1.** PAM50 molecular subtyping distribution in function of the nature of the breast tissue

Nature of the tissue	Molecular intrinsic subtype (PAM50)				
	Basal-like	HER2E	Luminal A	Luminal B	Normal breast-like
Healthy	0	0	89	0	3
Tumour-adjacent	0	0	101	0	3
Tumour	161	74	406	387	7

#### p53 status gene expression comparison analyses

p53-mutated status frequencies varied from 26.6% to 32.9% in function of the nature of the data (i.e. microarrays or RNAseq) and method of p53 status determination (i.e. IHC, GES and sequencing) (Table 2). These results are concordant with those observed in the literature for breast cancer (i.e. 20–35%) (24, 25). However, the lowest and significantly different frequency was observed for p53 status determination by means of p53 GES applied on microarray data (*P* < 0.0001). Other frequencies were comparable (*P* = 0.5121).

**Table 2.** Frequencies of p53 mutations in function of p53 mode of annotation and nature of the data

Data	TP53 status annotation	No	No (%) WT	No (%) MT
Microarrays	IHC	922	638 (69.2)	284 (30.8)
	Sequence-based	1980	1328 (67.1)	652 (32.9)
	GES	2728	2003 (73.4)	725 (26.6)
RNAseq	Sequence-based	1027	699 (68.1)	328 (31.9)

MT, mutated; No, number of; WT, wild type.

Expressions of the 31 probes and 26 genes belonging to p53 GES were concordant with their GES weights (−1 or +1) for IHC and sequence-based microarray data and for sequence-based RNAseq data (Supplementary Table S4).

Furthermore, expressions of the 47 probes and 40 genes belonging to proliferation GES showed that proliferation in p53-mutated tumours was higher than in p53 wild-type tumours irrespective of the method of p53 status determination in microarray data, and p53 status determination by sequencing in RNAseq data (Supplementary Table S5).

Immune response in function of p53 status was explored by means of eight immune response representative genes, 20 HLA genes and 12 immune checkpoint genes. Whatever the nature of the transcriptomic data (i.e. microarrays or RNAseq), a brief synthesis of these analyses demonstrates that immune response takes place in p53-mutated tumours whatever the method of p53 status determination (Supplementary Tables S6, S7 and S8).

#### Basal-like (PAM50) and/or TNBC (IHC)

**Figure 2.**
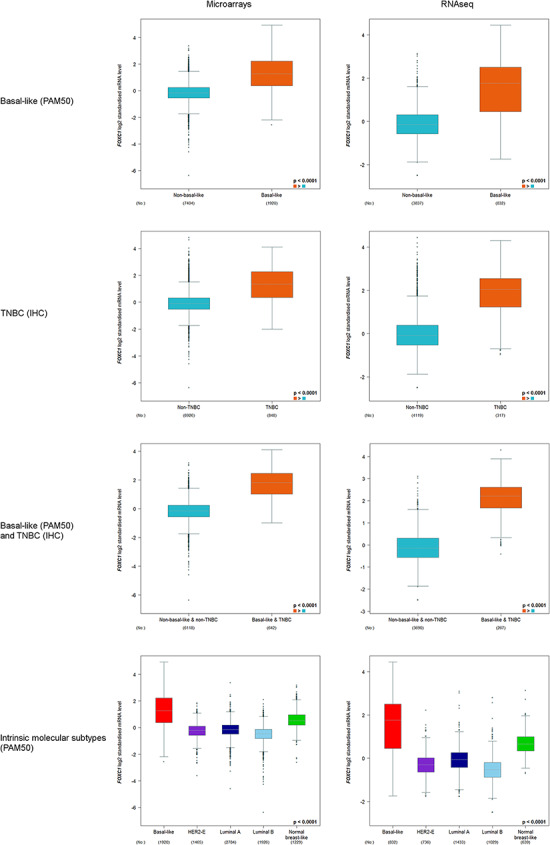
*FOXC1* gene expression analysis in basal-like (PAM50) and/or TNBC (IHC), and intrinsic molecular subtypes (PAM50).

*FOXC1* expression was always found to be significantly elevated in basal-like (PAM50) and/or TNBC(IHC) versus non-basal-like and/or non-TNBC, and in basal-like (PAM50) versus other intrinsic molecular subtypes (Figure 2).

#### TNBC subtypes

Transcriptomic TNBC data of eight Affymetrix® cohorts were selected (Supplementary Table S9). Unsupervised analysis followed by annotation using clinicopathological data, IHC markers and GES, separated TNBC into three subtypes: C1 [*n* = 169 (24.4%)], C2 [*n* = 252 (36.4%)] and C3 [*n* = 272 (39.2%)].

TNBC subtype profiles of the eight marker genes were concordant with what was expected (Table 3). Androgen signalling markers were highly expressed in C1, and immune-response markers were highly expressed in C3. *FOXC1*, which is a basal-like marker, displayed highest expression in C2 compared to C3, although these two subtypes are basal-like. This observation is likely in line with the fact that biological aggressiveness in C2 is more pronounced than in C3.

**Table 3.** Gene expression profiles of TNBC subtype–specific genes

Subtype specificity	Gene (median probe)	Biological process	TNBC profile
C1	*AR*	Androgen signalling	C1 > C2 = C3
	*FOXA1*	Androgen signalling	C1 > C2 = C3
C2	*FOXC1*	Development (basal-like marker)	C2 > C3 > C1
C3	*CD8A*	Immune response (T lymphocytes)	C3 > C1 > C2
	*IGKC*	Immune response (immunoglobulin)	C3 > C1 > C2
	*STAT1*	Immune response (interferon pathway)	C3 > C2 = C1
	*CD274 (PD-L1)*	Immune response (immune checkpoint)	C3 > C2 = C1
	*PDCD1 (PD1)*	Immune response (immune checkpoint)	C3 > C2 = C1

### Customized analysis

More than 100 000 differential gene expression analyses (20 000 genes × five splitting criteria) can be performed based on microarray data. This number increases to more than 180 000 (36 000 genes × five splitting criteria) by using RNAseq data.

**Figure 3.**
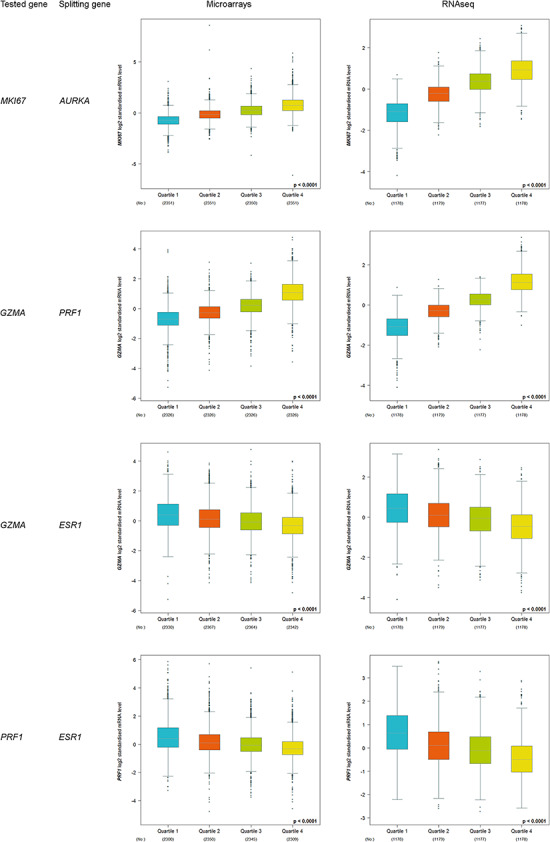
Customized expression analysis results of four demonstrative gene pairs (tested gene, splitting gene/quartile criterion): (*MKI67, AURKA*); (*GZMA, PRF1*); (*GZMA, ESR1*) and (*PRF1, ESR1*).

Increased kinetics, from Q1 to Q4, was observed for correlated proliferation and T-cell cytotoxicity genes (Figure 3). On the contrary, decreased kinetics was observed for T-cell cytotoxicity genes (*GZMA* and *PRF1*) in function of *ESR1* level. As expected, these results are concordant with the fact that immune response is triggered in ER-negative tumours, i.e. *ESR1*-low tumours.

## Discussion

From the very beginning, bc-genExMiner development is guided by one principle: to offer the most easy-to-use, reliable, complete, and biologically and clinically relevant web-based tool to breast cancer researchers and clinicians. Furthermore, a special effort was made to avoid ‘black box’ approach. The development of this new module was no exception with these guidelines; the handling of the expression module remains as simple as it ever was. Entry screens are not cluttered, analyses are performed in very few clicks and interpretation of the results is simple.

Different strategies have been applied in order to optimize the reliability of our web tool. First, strict inclusion criteria were used. Second, in order to limit normalization bias, normalization was carried out on the cohorts taken into account in specific analyses. Third, because no gold standard exists for intrinsic molecular subtyping and p53 status determinations, we proposed six modes of molecular subtyping and three robust subtyped cohorts (same annotation with different molecular subtype predictors) and three methods of p53 status determination. Fourth, analyses may be based on microarray or RNAseq pooled cohorts, or microarray unique cohort (METABRIC), or RNAseq unique cohorts (TCGA, SCAN-B). Concordant results based on different cohorts allow concluding that biological significance is robust. Fifth, each development of our web-based tool was validated by a large number of ‘biological tests’ whose aim was to prove that the pathobiological information of the gene expression data was present and not disrupted by the bioinformatics process (1, 2). All these validation test results confirmed that bc-GenExMiner bioinformatics process is globally neutral and that this web-based tool may be used for *in silico* validation or discovery purposes.

bc-GenExMiner belongs to the category of disease-associated web-based tools. Furthermore, it is considered as a complete tool. Indeed, users can test their genes of interest in multiple ways (expression, correlation and prognostic) by means of the same interface and know-how.

To the best of our knowledge, this new module includes at least two original kinds of gene expression analyses. Users can explore gene expression simultaneously in healthy mammary, tumour adjacent and tumour tissues. Here, healthy tissue is really a mammary tissue without any link with cancer. Biological kinetics observed between these three tissues (e.g. proliferation) demonstrated that tumour-adjacent tissue must not be assimilated to healthy tissue. Increasing (H < TA < T) and decreasing (H > TA > T) biological kinetics show that tumour-adjacent tissue has an intermediate pathological phenotype. Another originality of this new module is the possibility to explore gene expression in TNBC subtypes. We and others clearly showed that basal-like subtypes may be split into two distinct subtypes, notably in function of a pro-tumourigenic or an anti-tumourigenic immune response. Therefore, basal-like explorations have to take into account basal-like heterogeneity.

Finally, bc-GenExMiner continues to be actively developed. The updation of the gene names and inclusion of new cohorts are done regularly. By further increasing the number of patients, we will be able to explore gene expression in rare breast cancer cohorts for more specific investigations.

## Supplementary Material

baab007_Supp
