# Supplementary material for: bc-GenExMiner 4.5: new mining module computes breast cancer differential gene expression analyses
Source: Database (Oxford). 2021 Feb 18;2021:baab007. doi: 10.1093/database/baab007 (PMC7904047; doi:10.1093/database/baab007)
Supplement: baab007_Supp [file baab007_supp.zip › JEZEQUEL Supplementary Data.pdf]

Supplementary Table S1. Breast cancer microarray (**A**) and RNAseq (**B**) transcriptomic data included in bc-GenExMiner v4.5 database

A

Ver	Reference	No patients	Study code	Platform origin	Platform code	DNA chip	No. unique genes (2019)	Processing
1.0	Van de Vijver et al. 2002	295	Rosetta2002	Agilent	No code	25k oligo custom	14853	log2 ratio
1.0	Sotiriou et al. 2003	99	PNAS1732912100	NCI	No code	8k cDNA custom	4345	log2 ratio
1.0	Ma et al. 2004	59	GSE1378	Arcturus	GPL1223	22k oligo custom	14839	log2 ratio
1.0	Minn et al. 2005	82	GSE2603	Affymetrix®	GPL96	HG-U133A	12262	MAS5 and log2
1.0	Pawitan et al. 2005	159	GSE1456	Affymetrix®	GPL96 - GPL97	HG-U133A + B	18430	MAS5 and log2
1.0	Wang et al. 2005	286	GSE2034	Affymetrix®	GPL96	HG-U133A	12262	MAS5 and log2
1.0	Weigelt et al. 2005	50	GSE2741	Agilent	GPL1390	Human 1A oligo UNC custom	13980	log2 ratio
1.0	Bild et al. 2006	158	GSE3143	Affymetrix®	GPL91	HG-U95A v2	8767	MAS5 and log2
1.0	Chin et al. 2006	112	E_TABM_158	Affymetrix®	A-AFFY-76	HG-U133A v2	12262	MAS5 and log2
1.0	Ivshina et al. 2006	249	GSE4922	Affymetrix®	GPL96 - GPL97	HG-U133A + B	18430	MAS5 and log2
1.0	Desmedt et al. 2007	198	GSE7390	Affymetrix®	GPL96	HG-U133A	12262	MAS5 and log2
1.0	Loi et al. 2007	267	GSE6532	Affymetrix®	GPL96 - GPL97 - GPL570	HG U133A + B + P2	20542	MAS5 and log2
1.0	Minn et al. 2007	58	GSE5327	Affymetrix®	GPL96	HG-U133A	12262	MAS5 and log2
1.0	Naderi et al. 2007	135	E_UCON_1	Agilent	A-AGIL-14	Human 1A oligo G4110A	14258	log2 ratio
1.0	Anders et al. 2008	75	GSE7849	Affymetrix®	GPL91	HG-U95A v2	8767	MAS5 and log2
1.0	Chanrion et al. 2008	151	GSE9893	MLRG	GPL5049	Human 21k v12.0	15014	MAS5 and log2
1.0	Loi et al. 2008	77	GSE9195	Affymetrix®	GPL570	HG-U133P2	20542	MAS5 and log2
1.0	Calabrò et al. 2009	139	GSE10510	DKFZ	GPL6486	35k oligo	17807	log2 ratio
1.0	Jézéquel et al. 2009	252	GSE11264	UMGC-IRCNA	GPL4819	9k cDNA custom	1808	log2 ratio
1.1	Schmidt et al. 2008	200	GSE11121	Affymetrix®	GPL96	HG-U133A	12262	MAS5 and log2
1.1	Zhang et al. 2009	136	GSE12093	Affymetrix®	GPL96	HG-U133A	12262	MAS5 and log2
3.1	Chin et al. 2007	171	GSE8757	VUMC Microarray	GPL5737	Human 30K 60-mer oligo array	17782	log2 ratio
3.1	Zhou et al. 2007	54	GSE7378	Affymetrix®	GPL96	HG-U133A	12262	MAS5 and log2
3.1	Desmedt et al. 2009	55	GSE16391	Affymetrix®	GPL570	HG-U133P2	20542	MAS5 and log2
3.1	Jönsson et al. 2010	346	GSE22133	SweGene	GPL5345	H_v2.1.1 55K	9236	log2 ratio
3.1	Li et al. 2010	115	GSE19615	Affymetrix®	GPL570	HG-U133P2	20542	MAS5 and log2
3.1	Sircoulomb et al. 2010	55	GSE17907	Affymetrix®	GPL570	HG-U133P2	20542	MAS5 and log2
3.1	Buffa et al. 2011	216	GSE22219	Illumina	GPL6098	HumanRef-8 v1.0 expr-bc	15757	log2 ratio
3.1	Dedeurwaerder et al. 2011	85	GSE20711	Affymetrix®	GPL570	HG-U133P2	20542	MAS5 and log2
3.1	Filipits et al. 2011	277	GSE26971	Affymetrix®	GPL96	HG-U133A	12262	MAS5 and log2
3.1	Hatzis et al. 2011	309	GSE25055	Affymetrix®	GPL96	HG-U133A	12262	MAS5 and log2
3.1	Kao et al. 2011	296	GSE20685	Affymetrix®	GPL570	HG-U133P2	20542	MAS5 and log2
3.1	Sabatier et al. 2011	239	GSE21653	Affymetrix®	GPL570	HG-U133P2	20542	MAS5 and log2
3.1	Wang et al. 2011	149	GSE16987	Illumina	GPL6104	HumanRef-8 v2.0 expr-bc	17132	log2 ratio
3.1	Kuo et al. 2012	51	GSE33926	Agilent	GPL7264	Human 1A Microarray (V2) G4110B	16641	log2 ratio
3.1	Nagalla et al. 2013	41	GSE45255	Affymetrix®	GPL96	HG-U133A	12262	MAS5 and log2
4.3	expO et al. 2005	298	GSE2109	Affymetrix®	GPL570	HG-U133P2	20542	MAS5 and log2
4.3	Yau et al. 2007	47	GSE8193	Affymetrix®	GPL96	HG-U133A	12262	MAS5 and log2
4.3	Parris et al. 2010	94	GSE20462	Illumina	GPL6947	HumanHT-12 V3.0	19016	Quantile norm. and log2
4.3	Symmans et al. 2010	43	GSE17705	Affymetrix®	GPL96	HG-U133A	12262	MAS5 and log2
4.3	Heikkinen et al. 2011	174	GSE24450	Illumina	GPL6947	HumanHT-12 V3.0	19016	Quantile norm. and log2
4.3	Sabatier et al. 2011	71	GSE31448	Affymetrix®	GPL570	HG-U133P2	20542	MAS5 and log2
4.3	Curtis et al. 2012	1980	METABRIC	Illumina	GPL6947	HumanHT-12 V3.0	18025	Quantile norm. and log2
4.3	Guedj et al. 2012	536	E_MTAB_365	Affymetrix®	GPL570	HG-U133P2	20542	MAS5 and log2
4.3	Servant et al. 2012	343	GSE30682	Illumina	GPL6884	HumanWG-6 v3.0	19016	Quantile norm. and log2
4.3	Clarke et al. 2013	104	GSE42568	Affymetrix®	GPL570	HG-U133P2	20542	MAS5 and log2
4.3	Larsen et al. 2013	183	GSE40115	Agilent	GPL15931	SurePrint G3 Human GE 8x60K	20118	log2 ratio
4.3	Castagnoli et al. 2014	53	GSE55348	Illumina	GPL14951	HumanHT-12 WG-DASL V4.0 R2	19459	Quantile norm. and log2
4.3	Fumagalli et al. 2014	56	GSE43358	Affymetrix®	GPL570	HG-U133P2	20542	MAS5 and log2
4.3	Merdad et al. 2014	45	GSE36295	Affymetrix®	GPL6244	Gene 1.0 ST	20251	rma-gene-level
4.3	Terunuma et al. 2014	55	GSE37751	Affymetrix®	GPL6244	Gene 1.0 ST	20251	rma-gene-level
4.3	Burstein et al. 2015	66	GSE76274	Affymetrix®	GPL570	HG-U133P2	20542	MAS5 and log2
4.3	Michaut et al. 2016	104	GSE68057	Agilent	GPL20078	Agendia32627 DPv1.14 SCFGplus	20209	Quantile norm. and log2
4.3	Biermann et al. 2017	53	GSE97177	Illumina	GPL6947	HumanHT-12 V3.0	19016	Quantile norm. and log2
4.5	Bos et al. 2009	204	GSE12276	Affymetrix®	GPL570	HG-U133P2	20542	MAS5 and log2
4.5	Silver et al. 2010	75	GSE18864	Affymetrix®	GPL570	HG-U133P2	20542	MAS5 and log2
4.5	Burstein et al. 2015	198	GSE76124	Affymetrix®	GPL570	HG-U133P2	20542	MAS5 and log2
4.5	Jézéquel et al. 2015	107	GSE58812	Affymetrix®	GPL570	HG-U133P2	20542	MAS5 and log2
4.5	Jézéquel et al. 2019	131	GSE83937	Affymetrix®	GPL570	HG-U133P2	20542	MAS5 and log2

Ver: version number of bc-GenExMiner; No: number of

B

Ver	Reference	No patients	Study code	Platform origin	Platform code	DNA chip	No. unique genes (2019)	Processing	Analysis
4.3	TCGA et al. 2012	1034	TCGA	RNAseq	Illumina	HiSeq	36237	FPKM and log2	All except « Nature of the tissue »
4.3	Brueffer et al. 2018	405	SCAN-B / GSE81538	Illumina	GPL11154	HiSeq 2000	18628	FPKM and log2	
4.3	Saal et al. 2018	3273	SCAN-B / GSE96058	Illumina	GPL11154	HiSeq 2000	23638	FPKM and log2	
4.5	TCGA et al. 2012	1142	TCGA	RNAseq	Illumina	HiSeq	22444	TPM and log2	« Nature of the tissue »
4.5	GTEx et al. 2019	92	GTEx	RNAseq	Illumina	HiSeq	22444	TPM and log2	

Ver: version number of bc-GenExMiner; No: number of

Supplementary Table S2. Breast cancer clinocopathological characteristics associated with microarray (**A**) and RNAseq (**B**) transcriptomic data included in bc-GenExMiner v4.5 database.

A

Ver	Reference	No patients	ER[a]	PR[a]	HER2[a]	Nodal status	Histo. type	SBR	NPI	AOL	Age diagn.	P53			SSPs	SCMs	Event status		
												IHC	Seq	GES			DMFS	OS	DFS
1.0	Van de Vijver et al. 2002	295	295	41	-	295	-	41	40[c]	-	41	-	-	-	295	295	101	79	122
1.0	Sotiriou et al. 2003	99	99	-	-	99	99	99	99	99	99	-	-	-	90	99	30	45	53
1.0	Ma et al. 2004	59	59	59	55	52	59	59	52	52	59	-	-	-	59	59	-	-	27
1.0	Minn et al. 2005	82	82	82	76	82	-	-	-	-	82	-	-	-	82	82	27	-	27
1.0	Pawitan et al. 2005	159	159[b]	-	-	-	-	147	-	-	-	-	-	159	159	159	40	40	50
1.0	Wang et al. 2005	286	286	-	-	286	-	-	-	-	-	-	-	-	286	286	107	-	107
1.0	Weigelt et al. 2005	50	50	-	-	50	-	50	21[c]	-	50	-	-	-	50	50	13	10	13
1.0	Bild et al. 2006	158	158[b]	-	-	-	-	-	-	-	-	-	-	-	158	158	-	50	50
1.0	Chin et al. 2006	112	112	112	78	112	-	107	46[c]	-	112	80	-	-	99	112	21	35	42
1.0	Ivshina et al. 2006	249	245	0	0	240	-	249	159[c]	-	249	247	-	249	249	249	0	0	89
1.0	Desmedt et al. 2007	198	198	0	0	198	184	196	196	196	198	-	-	-	198	198	62	56	91
1.0	Loi et al. 2007	267	261	87	0	261	0	208	123[c]	0	267	-	-	267	266	267	66	0	88
1.0	Minn et al. 2007	58	58	0	0	0	0	0	0	0	0	-	-	-	58	58	11	0	11
1.0	Naderi et al. 2007	135	133	0	0	129	0	134	129	128	135	-	-	-	127	135	0	47	65
1.0	Anders et al. 2008	75	71	70	0	75	74	64	64	61	75	-	-	-	75	75	14	0	14
1.0	Chanrion et al. 2008	151	139	139	0	146	139	144	134	124	151	-	-	-	139	151	46	41	55
1.0	Loi et al. 2008	77	77	77	0	77	0	58	30[c]	0	77	-	-	77	77	77	10	0	13
1.0	Calabrò et al. 2009	139	136	136	49	103	0	0	0	0	139	-	-	-	116	139	0	63	96
1.0	Jézéquel et al. 2009	252	239	236	203	252	0	252	252	252	252	-	-	-	0	0	65	47	68
1.1	Schmidt et al. 2008	200	200[b]	0	0	200	0	200	200	0	0	-	-	-	200	200	46	0	46
1.1	Zhang et al. 2009	136	136	136	0	136	0	0	0	0	0	-	-	-	136	136	20	0	20
3.1	Chin et al. 2007	171	170	0	0	170	0	170	170	0	171	-	-	-	152	171	38	57	56
3.1	Zhou et al. 2007	54	54	0	0	54	0	0	0	0	54	-	-	-	54	54	9	0	9
3.1	Desmedt et al. 2009	55	55	55	45	55	0	55	0	0	55	-	-	55	55	55	0	0	55
3.1	Jönsson et al. 2010	346	335	332	0	0	0	226	0	0	0	-	-	-	346	0	0	151	151
3.1	Li et al. 2010	115	115	115	115	115	103	115	64[c]	0	115	-	-	115	115	115	14	0	14
3.1	Sircoulomb et al. 2010	55	47	47	37	45	33	47	0	0	49	29	-	55	55	55	17	0	17
3.1	Buffa et al. 2011	216	216	0	0	216	0	191	191	0	216	0	-	0	216	216	82	0	82
3.1	Dedeurwaerder et al. 2011	85	84	0	85	85	85	85	29[c]	0	85	0	-	85	85	85	0	0	36
3.1	Filipits et al. 2011	277	277	0	277	0	0	0	0	0	0	0	-	0	276	277	58	0	58
3.1	Hatzis et al. 2011	309	304	303	309	309	0	286	0	0	309	0	-	0	309	309	65	0	65
3.1	Kao et al. 2011	296	296[b]	0	0	296	0	0	0	0	296	0	-	296	296	296	63	62	73
3.1	Sabatier et al. 2011	239	237	237	224	233	211	233	0	0	238	175	-	239	239	239	0	0	74
3.1	Wang et al. 2011	149	149	149	149	148	147	149	148	0	149	0	-	0	149	149	0	0	10
3.1	Kuo et al. 2012	51	51	51	51	51	51	47	0	0	51	0	-	0	51	51	12	0	12
3.1	Nagalla et al. 2013	41	40	38	39	41	0	39	39	36	41	0	-	0	41	41	14	10	14
4.3	expO et al. 2005	298	210	209	198	257	289	252	39[c]	0	298	0	-	298	298	298	0	0	0
4.3	Yau et al. 2007	47	47	47	0	43	0	0	0	0	47	0	-	0	47	47	0	0	0
4.3	Parris et al. 2010	94	94	94	94	94	80	75	75	0	93	0	-	0	93	94	0	44	45
4.3	Symmans et al. 2010	43	43	0	0	42	0	0	0	0	0	0	-	0	43	43	71	0	71
4.3	Heikkinen et al. 2011	174	0	0	0	0	0	0	0	0	0	0	-	0	172	174	34	27	34
4.3	Sabatier et al. 2011	71	71	71	19	26	0	0	0	0	44	0	-	71	71	71	0	0	0
4.3	Curtis et al. 2012	1980	1937	1980	1980	1980	1830	1892	1875	0	1980	0	1980	0	1978	1980	602	1143	1235
4.3	Guedj et al. 2012	536	515	514	390	438	427	517	0	0	523	239	0	536	536	536	119	0	119
4.3	Servant et al. 2012	343	0	0	0	337	318	339	0	0	343	97	0	0	343	343	119	0	119
4.3	Clarke et al. 2013	104	101	0	0	104	0	104	45[c]	0	104	0	0	104	104	104	48	35	48
4.3	Larsen et al. 2013	183	183	183	183	0	169	157	0	0	183	0	0	0	182	183	0	0	0
4.3	Castagnoli et al. 2014	53	53	53	53	53	0	53	0	0	53	0	0	0	53	53	23	0	23
4.3	Fumagalli et al. 2014	56	56	56	56	54	52	56	54	0	55	0	0	56	56	56	0	0	0
4.3	Merdad et al. 2014	45	38	38	38	0	40	38	0	0	45	0	0	0	45	45	0	0	0
4.3	Terunuma et al. 2014	55	55	12	12	55	0	48	244[c]	0	55	55	0	0	55	55	0	19	19
4.3	Burstein et al. 2015	66	66	66	49	0	64	47	0	0	63	0	0	66	66	66	0	0	0
4.3	Michaut et al. 2016	104	88	92	89	104	95	96	83	0	103	0	0	0	104	104	26	20	32
4.3	Biermann et al. 2017	53	52	52	53	42	0	0	0	0	53	0	0	0	53	53	0	0	0
4.5	Bos et al. 2009	204	56	56	56	0	0	0	0	0	0	0	0	0	0	0	0	0	0
4.5	Silver et al. 2010	75	35	35	35	0	0	0	0	0	0	0	0	0	0	0	0	0	0
4.5	Burstein et al. 2015	198	198	198	198	0	0	0	0	0	0	0	0	0	0	0	0	0	0
4.5	Jézéquel et al. 2015	107	107	107	107	0	0	0	0	0	0	0	0	0	0	0	0	0	0
4.5	Jézéquel et al. 2019	131	131	131	131	0	0	0	0	0	0	0	0	0	0	0	0	0	0
	Total	10716	9759	6496	5533	8240	4549	7325	4381	948	7857	922	1980	2728	9657	9403	2093	2081	3618

[a] ER Oestrogen receptor, progesterone receptor (PR) and HER2 status determined by immunohistochemistry (IHC)
[b] ER status determined by means of genomics data (Affymetrix® probe: 205225_at) in case of a lack of IHC data
[c] NPI score could be computed only for node negative patients

Ver: version number of bc-GenExMiner; No: number of; Histo. Type: histological type; SBR: Scarff-Bloom and Richardson grade; NPI: Nottingham Prognostic Index; AOL: Adjuvant! Online; Age diagn.: age at diagnosis; IHC: immunohistochemistry; Seq: sequence-based; GES: gene expression signature; SSPs: single sample predictors; SCMs: subtype clustering models; DMFS: distant metastasis-free survival (first pejorative event represented by distant relapse); OS: overall survival (first pejorative event represented by death); DFS: disease-free survival (first pejorative event represented by any relapse or death); -: no data

B

Ver	Reference	No patients	ER[a]	PR[a]	HER2[a]	Nodal status	Histo. type	SBR	NPI	AOL	Age diagn.	P53			SSPs	SCMs	Event status		
												IHC	seq	GES			DMFS	OS	DFS
Healthy																			
4.5	GTEx et al. 2019	92	-	-	-	-	-	-	-	-	-	-	-	-	-	-	-	-	-
Tumour-adjacent																			
4.5	TCGA et al. 2012	104	-	-	-	-	-	-	-	-	-	-	-	-	-	-	-	-	-
Tumour																			
4.3	TCGA et al. 2012	1034	988	985	689	881	989	-	-	-	1033	-	1031	-	1034	1034	-	99	99
4.3	Brueffer et al. 2018	405	405	405	405	-	-	405	-	-	-	-	-	-	405	405	-	-	-
4.3	Saal et al. 2018	3273	3073	2940	3151	3180	-	3212	3114	-	3273	-	-	-	3270	3273	-	336	336
	Total	4712	4466	4330	4245	4061	989	3617	3114	-	4306	-	2062	-	4709	4712	-	435	435

[a] ER Oestrogen receptor, progesterone receptor (PR) and HER2 status determined by immunohistochemistry (IHC)

Ver: version number of bc-GenExMiner; No: number of; Histo. Type: histological type; SBR: Scarff-Bloom and Richardson grade; NPI: Nottingham Prognostic Index; AOL: Adjuvant! Online; Age diagn.: age at diagnosis; IHC: immunohistochemistry; Seq: sequence-based; GES: gene expression signature; SSPs: single sample predictors; SCMs: subtype clustering models; DMFS: distant metastasis-free survival (first pejorative event represented by distant relapse); OS: overall survival (first pejorative event represented by death); DFS: disease-free survival (first pejorative event represented by any relapse or death); -: no data

Supplementary Table S3. Continuous score gene-expression signatures analyses results in function of three types of breast tissues

GES name	*p* value	*p* value			Results
		H vs TA	H vs T	TA vs T	
**Metabolism**					
Glycolysis	< 0.0001	0.8133	< 0.0001	< 0.0001	H = TA < T
IRGS	< 0.0001	0.3907	< 0.0001	< 0.0001	H = TA < T
**Critical biological pathways in cancer**					
CIN	< 0.0001	0.0004	< 0.0001	< 0.0001	H < TA < T
E2F3	< 0.0001	0.0123	< 0.0001	0.2855	H < TA = T
MYC	< 0.0001	< 0.0001	< 0.0001	< 0.0001	H < TA < T
PNI	< 0.0001	0.0534	< 0.0001	< 0.0001	H = TA < T
Proliferation	< 0.0001	0.0001	< 0.0001	< 0.0001	H < TA < T
TGFβ	< 0.0001	< 0.0001	< 0.0001	0.0254	H > TA > T
Wound-response	< 0.0001	< 0.0001	< 0.0001	< 0.0001	H < TA < T
**Prognosis**					
38-GES	< 0.0001	0.7814	< 0.0001	< 0.0001	H = TA < T
70-GES	< 0.0001	0.5975	< 0.0001	< 0.0001	H = TA < T
GGI	< 0.0001	0.0219	< 0.0001	< 0.0001	H < TA < T

GES: gene expression signature; H: breast healthy tissue; TA: breast tumour-adjacent tissue; T: breast tumour tissue; IRGS: iron regulatory gene signature; CIN: chromosomal instability; E2F3: E2F3 transcription factor 3; MYC: MYC proto-oncogene BHLH transcription factor; PNI: perineural invasion; TGFβ: transforming growth factor beta, GGI genomic grade index

Supplementary Table S4. Comparisons of gene expressions of genes included in p53 gene expression signature in function of p53 status (wild type or mutated) determined by immunohistochemistry or sequencing. Analyses are carried out using microarray and RNAseq data

Affymetrix probe set ID	Gene	GES gene weight	Microarrays		RNAseq
			Affymetrix	METABRIC	TCGA
			IHC	Seq-based	Seq-based
217889_s_at	*CYBRD1*	1	>	>	>
243929_at	No gene	1	>	NA	NA
229975_at	*BMPR1B*	1	>	>	>
223864_at	*ANKRD30A*	1	>	>	>
227081_at	*DNALI1*	1	>	>	>
215014_at	*KCND3*	1	>	=	>
206869_at	*CHAD*	1	>	>	>
221585_at	*CACNG4*	1	>	>	>
205440_s_at	*NPY1R*	1	>	>	>
228969_at	*AGR2*	1	>	>	>
212949_at	*NCAPH*	-1	<	=	<
226067_at	*BPIFB1*	1	>	>	>
232855_at	No gene	1	>	NA	NA
221520_s_at	*CDCA8*	-1	<	<	<
205472_s_at	*DACH1*	1	>	>	>
205186_at	*DNALI1*	1	>	>	>
221275_s_at	No gene	1	>	NA	NA
229030_at	*CAPN8*	1	>	>	>
233413_at	No gene	1	>	NA	NA
203438_at	*STC2*	1	>	>	>
230378_at	*SCGB3A1*	1	>	>	>
238581_at	*GBP5*	-1	<	<	<
235343_at	*VASH2*	-1	<	<	<
229150_at	No gene	1	>	NA	NA
205734_s_at	*AFF3*	1	>	>	>
214079_at	*DHRS2*	1	>	>	>
238746_at	*PXMP4*	1	>	>	>
204623_at	*TFF3*	1	>	>	>
230863_at	*LRP2*	1	>	>	>
215047_at	*TRIM58*	1	>	NA	NA
201710_at	*MYBL2*	-1	<	=	<

GES: gene expression signature; IHC: immunohistochemistry; Seq-based: sequence-based; <: p53 wild type < p53 mutated ($p < 0.05$); >: p53 wild type > p53 mutated ($p < 0.05$); =: p53 wild type = p53 mutated ($p \geq 0.05$); NA: no analysis

Supplementary Table S5. Comparisons of gene expressions of genes included in proliferation gene expression signature in function of p53 status (wild type or mutated) determined by immunohistochemistry or sequencing. Analyses are carried out using microarray and RNAseq data

Affymetrix probe set ID	Gene	GES gene weight	Microarrays			RNAseq
			Affymetrix		METABRIC	TCGA
			IHC	GES	Seq-based	Seq-based
206102_at	*GINS1*	1	<	<	=	<
209172_s_at	*CENPF*	1	<	<	<	<
203418_at	*CCNA2*	1	<	<	<	<
204026_s_at	*ZWINT*	1	<	<	<	<
203213_at	*CDK1*	1	<	<	<	<
203214_x_at	*CDK1*	1	<	<	<	<
210559_s_at	*CDK1*	1	<	<	<	<
204170_s_at	*CKS2*	1	<	<	<	<
204092_s_at	*AURKA*	1	<	<	<	<
208079_s_at	*AURKA*	1	<	<	<	<
204092_s_at	*AURKA*	1	<	<	<	<
213226_at	*CCNA2*	1	<	<	<	<
202095_s_at	*BIRC5*	1	<	<	<	<
202613_at	*CTPS1*	1	<	<	<	<
201291_s_at	*TOP2A*	1	<	<	<	<
201292_at	*TOP2A*	1	<	<	<	<
222039_at	*KIF18B*	1	<	<	<	<
204822_at	*TTK*	1	<	<	<	<
202954_at	*UBE2C*	1	<	<	<	<
203755_at	*BUB1B*	1	<	<	NA	<
202705_at	*CCNB2*	1	<	<	<	<
204962_s_at	*CENPA*	1	<	<	<	<
209642_at	*BUB1*	1	<	<	<	<
202870_s_at	*CDC20*	1	<	<	<	<
209408_at	*KIF2C*	1	<	<	<	<
221520_s_at	*CDCA8*	1	<	<	<	<
218039_at	*NUSAP1*	1	<	<	<	<
206364_at	*KIF14*	1	<	<	<	<
204641_at	*NEK2*	1	<	<	<	<
207828_s_at	*CENPF*	1	<	<	<	<
219918_s_at	*ASPM*	1	<	<	<	<
204444_at	*KIF11*	1	<	<	<	<
218542_at	*CEP55*	1	<	<	<	<
213008_at	*FANCI*	1	<	<	<	<
213007_at	*FANCI*	1	<	<	<	<
218009_s_at	*PRC1*	1	<	<	<	<
210052_s_at	*TPX2*	1	<	<	<	<
203764_at	*DLGAP5*	1	<	<	<	<
214710_s_at	*CCNB1*	1	<	<	<	<
218355_at	*KIF4A*	1	<	<	<	<
202580_x_at	*FOXM1*	1	<	<	<	<
221436_s_at	*CDCA3*	1	<	<	<	<
218755_at	*KIF20A*	1	<	<	<	<
218663_at	*NCAPG*	1	<	<	<	<
219148_at	*PBK*	1	<	<	<	<
218585_s_at	*DTL*	1	<	<	<	<
218726_at	*HJURP*	1	<	<	<	<

GES: gene expression signature; IHC: immunohistochemistry; Seq-based: sequence-based; <: p53 wild type < p53 mutated ($p < 0.05$); =: p53 wild type = p53 mutated ($p \geq 0.05$); NA: no analysis

Supplementary Table S6. Comparisons of representative immune response gene expressions in function of p53 status (wild type or mutated) determined by immunohistochemistry, gene expression signature or sequencing. Analyses are carried out using microarray and RNAseq data

Gene	Microarrays			RNAseq
	All		METABRIC	TCGA
	IHC	GES	Seq-based	Seq-based
CD8A	<	<	<	<
GZMA	<	<	<	<
GZMB	<	<	<	<
IGHM	<	<	NA	<
IGKC	<	<	<	<
MS4A1 (CD20)	<	<	<	<
PRF1	<	<	<	<
STAT1	<	<	<	<

IHC: immunohistochemistry; GES: gene expression signature; Seq-based: sequence-based; <: p53 wild type < p53 mutated ($p < 0.05$); NA: no analysis

Supplementary Table S7. Comparisons of HLA gene expressions in function of p53 status (wild type or mutated) determined by immunohistochemistry, gene expression signature or sequencing. Analyses are carried out using microarray and RNAseq data

Gene	Microarrays			RNAseq
	All IHC	GES	METABRIC Seq-based	TCGA Seq-based
HLA-DRA	=	<	<	<
HLA-DMA	<	<	<	<
HLA-DRB6	=	<	NA	<
HLA-DQA1	=	<	<	<
HLA-E	<	<	<	<
HLA-DPB1	=	<	<	=
HLA-DPA1	=	<	<	=
HLA-H	NA	NA	<	<
HLA-G	<	<	<	<
HLA-DOA	=	<	<	=
HLA-DOB	<	<	<	<
HLA-DQA2	=	NA	<	=
HLA-DMB	<	<	<	<
HLA-A	<	<	<	<
HLA-F	<	<	<	<
HLA-B	<	<	<	<
HLA-DRB1	<	<	=	<
HLA-DQB1	<	<	=	<
HLA-C	<	<	<	<
HLA-DRB4	=	<	<	NA

IHC: immunohistochemistry; GES: gene expression signature; Seq-based: sequence-based ; <: p53 wild type < p 53 mutated ($p < 0.05$); =: p53 wild type = p53 mutated ($p \geq 0.05$); NA: no analysis

Supplementary Table S8. Comparisons of immune checkpoint gene expressions in function of p53 status (wild type or mutated) determined by immunohistochemistry, gene expression signature or sequencing. Analyses are carried out using microarray and RNAseq data

Gene	Microarrays			RNAseq
	All		METABRIC	TCGA
	IHC	GES	Seq-based	Seq-based
BTLA	<	<	<	<
CD27	<	<	<	<
CD80	<	<	<	<
CD86	<	<	<	<
CD274 (PD-L1)	<	<	<	<
CTLA4	<	<	<	<
IDO1	<	<	<	<
IDO2	<	<	<	<
LAG3	<	<	<	<
PDCD1 (PD1)	<	<	<	<
PDCD1LG2 (PD-L2)	<	<	<	<
TIGIT	<	<	<	<

IHC: immunohistochemistry; GES: gene-expression signature; Seq-based: sequence-based; <: p53 wild type < p53 mutated ($p < 0.05$)

Supplementary Table S9. TNBC cohorts used for gene expression analysis in TNBC subtypes

Study code	DNA chip	TNBC patients	Reference
E-MTAB-365	Affymetrix U133P2	51	(S1)
GSE12276	Affymetrix U133P2	56	(S2)
GSE18864	Affymetrix U133P2	35	(S3)
GSE19615	Affymetrix U133P2	28	(S4)
GSE21653	Affymetrix U133P2	87	(S5)
GSE58812	Affymetrix U133P2	107	(S6)
GSE76124	Affymetrix U133P2	198	(S7)
GSE83937	Affymetrix U133P2	131	(S8)

References

S1. Guedj,M., Marisa,L., de Reynies,A., *et al*. (2011) A refined molecular taxonomy of breast cancer. *Oncogene*, **31**, 1196-1206.
S2. Bos,P.D., Zhang,X.H., Nadal,C., *et al*. (2009) Genes that mediate breast cancer metastasis to the brain. *Nature*, **459**, 1005-1009.
S3. Silver,D.P., Richardson,A.L., Eklund,A.C., *et al*. (2010) Efficacy of neoadjuvant Cisplatin in triple-negative breast cancer. *J. Clin. Oncol.*, **28**, 1145-1153.
S4. Li,Y., Zou,L., Li,Q., *et al*. (2010) Amplification of LAPTM4B and YWHAZ contributes to chemotherapy resistance and recurrence of breast cancer. *Nat. Med.*, **16**, 214-218.
S5. Sabatier,R., Finetti,P., Cervera,N., *et al*. (2011) A gene expression signature identifies two prognostic subgroups of basal breast cancer. *Breast Cancer Res. Treat.*, **126**, 407-420.
S6. Jézéquel,P., Loussouarn,D., Guérin-Charbonnel,C., *et al*. (2015) Gene-expression molecular subtyping of triple-negative breast cancer tumours: importance of immune response. *Breast Cancer Res.*, **17**, 43.
S7. Burstein,M.D., Tsimelzon,A., Poage,G.M., *et al*. (2015) Comprehensive genomic analysis identifies novel subtypes and targets of triple-negative breast cancer. *Clin. Cancer Res.*, **21**, 1688-1698.
S8. Jézéquel,P., Kerdraon,O., Hondermarck,H., *et al*. (2019) Identification of three subtypes of triple-negative breast cancer with potential therapeutic implications. *Breast Cancer Res.*, **21**, 65.
